# Case of a Considerable Effusion of Bile into the Cavity of the Abdomen, in Consequence of a Fall; with the Appearances on Dissection, and Some Additional Remarks

**Published:** 1785

**Authors:** 


VI. *Caſe of a conſiderable Effuſion of Bile into the Cavity of the Abdomen, in conſequence of a Fall; with the Appearances on Diſſection, and ſome additional Remarks. By* Thomas Skeete, *M. D. Phyſician in London.*

WILLIAM Carter, fourteen years of age, and healthy, after having received a fall from a mulberry tree early on the morning of the 11th of Auguſt, 1783, by which he ſtruck his belly forcibly on the ground, was admitted a patient in Guy's hoſpital the ſame day at noon, under the care of Dr. Saunders, who firſt ſuggeſted to me the propriety of making the caſe known to the public, and kindly gave me his permiſſion to do whatever I ſhould think proper with it.

The ſymptoms at the time of admiſſion were, conſiderable pain in the abdomen, increaſed on preſſure, ſlight nauſea, and quickneſs of the pulſe. He had vomited ſeveral times ſoon after the accident, and ſome blood had been taken from his arm before he was brought to the hoſpital. At this viſit he was ordered to loſe ten ounces more of blood, to take a purging draught of oil, manna, and ſalts, with an opiate after its operation, and to have emollient clyſters occaſionally.

On the day following the pain had increaſed, and he complained of thirſt; the pulſe was quick and ſmall, and the blood ſizy; but there was ſcarcely any tenſion of the abdomen, and ſeveral ſtools had been procured. The blood-letting, purging draught, and clyſters were repeated, and he was put into the warm bath. The morning afterwards he was much relieved, and on the fourth day from the accident was ſo well as to be out of bed, and walk about the room without inconvenience; but on the fifth day he again complained of the pain, and on the ſixth was ſeized with chillineſs and other ſymptoms of fever, with an increaſe of pain, eſpecially on preſſure, accompanied by ſome degree of tenſion of the abdomen, and coſtiveneſs. The clyſters and oily purging draught were repeated, and the abdomen was ordered to be well fomented.

His complaints increaſed during the two ſucceeding days; the pain became exceedingly acute on preſſure, and the pulſe quicker, with reſtleſſneſs and thirſt; ſo that on the eighth day he was directed to loſe eight ounces of blood, to take ſaline draughts with ſoluble tartar every fourth hour, and to continue the clyſters: a warm ſtimulating plaſter was likewiſe applied over the whole of the abdomen; and whenever he expreſſed an inclination for drink, he was allowed barley water *ad libitum*. He remained nearly in the ſame condition on the ninth as on the day before, except that ſtools had been procured — the blood was again found ſizy. The pain continued acute on the tenth day, particularly about the ſcrobiculus cordis and right hypochondrium, accompanied by fever, and enlargement of the abdomen; to which a large bliſter was applied. On the ſucceeding day day he was wonderfully relieved, and on the twelfth and thirteenth continued free from pain; but the abdomen increafed in fize, fluctuation became evident, and he paffed but little urine. During the next five days there was no material alteration; his breathing was now and then difficult, but as foon as ftools were procured he was eafier, and in good fpirits. The urine was high coloured, and in fmall quantity; and, although his appetite was not bad, he was confiderably emaciated. Two grains of dried fquills were prefcribed three times a day. This remedy produced no particular effect; fo that in the courfe of the five fucceeding days the abdomen continued to enlarge. The oily purging draught having loft its efficacy, coftivenefs was relieved by an infufion of fenna with fyrup of buckthorn. He was likewife directed to take of jalap and crem. tart. āā gr. xv. twice a week.

On the twenty-fourth day from the accident the difficulty of breathing had increafed, the pulfe was very quick, and he was much emaciated, efpecially about the upper extremities; his countenance was alfo pale, the abdomen greatly diftended, and the fluctuation fenfible in every part. The operation of the paracentefis tesis was performed, and fixteen pints of yellow fluid evacuated, which anfwered in every refpect to bile, containing apparently a very large proportion of that fluid. It was bitter, became green on the addition of acids, and when evaporated to drynefs, deflagrated with nitre. Upon inquiry, it was afcertained that his ftools had been white and of a clayey confiftence from the time of the fall. An anodyne draught was adminiftered after the operation, and a mixture of confect. cardiac. and aq. menth. given occafionally. He was free from pain for the two next days, except a little on the night after the tapping; the quicknefs of his pulfe abated, his fpirits were good, and his appetite improved. There was no enlargement whatever of the abdomen, nor the flighteft traces of bile in the circulation; but on the third day after the operation, and the twenty-feventh from the beginning, the fymptoms again made their appearance, which occafioned great dejection of mind, as he had flattered himfelf there would be no return of the complaint. On the twenty-eighth day half an ounce of vinous tincture of rheubarb was ordered to be taken occafionally to obviate coftivenefs. On the twenty-ninth, thirtieth, and thirtythirty-firſt, he was relieved by the tincture; but the abdomen increaſed in ſize, the urine was ſcanty, high coloured, and depoſited a copious ſediment. The five ſucceeding days the ſymptoms were nearly as before, the abdomen conſiderably enlarged, the pulſe quick and weak, with low ſpirits and great emaciation. He was anxious, notwithſtanding, to be tapped a ſecond time.

On the thirty-ſeventh day from the accident, and the fifteenth from the operation, an attempt was again made to diſcharge the accumulated fluid, but without ſucceſs, although the inſtrument was introduced both on the left and right ſides of the abdomen, in the uſual places; from the former about one ſpoonful of the bilious diſcharge was obtained; and from the other a ſmall quantity of blood only. It was obſervable, on a more minute examination, that the belly was not ſo tenſe, nor the fluctuation by any means ſo evident as before the firſt tapping. The next day his pulſe and reſpiration were remarkably quick, the abdomen extremely ſore, ſcarcely bearing the weight of a finger, his appetite totally loſt, his countenance ſunk, and his ſpirits depreſſed. The common ſaline draughts were preſcribed three times a day, with with five drops of laudanum in each, and twelve additional drops of it at night; likewife he was fomented, and continued to take the tincture of rhubarb. On the day following there was lefs pain and forenefs, and the frequency of his pulfe was fomewhat diminifhed. The medicines were repeated. He became worfe, however, during the two fucceeding days; his breathing was fhort and difficult, the pulfe quick and weak, and the pain uncommonly diftreffing. He died early on the next morning, after having furvived the unfortunate accident fix weeks.

*Appearances on Diffection.*

An opening was firft made into the left fide, near the part where the trocar had been introduced, but this, and a larger incifion feveral inches higher, gave difcharge only to a fmall quantity of pus. On making an incifion, however, from the fcrobiculus cordis to the navel, and paffing the knife through the peritonæum, near to the former, a confiderable quantity of a dark bilious fluid fuddenly made its efcape. When this was difcharged, the contents of the abdomen were farther expofed, and exhibited the moft confufed appearance that it is poffible to to conceive. The right hypochondrium, inſtead of containing the liver, formed a large cavity, in which a great quantity of the fluid ſtill remained; this, when removed and added to the former portion, meaſured between two and three gallons. The liver had in fact been preſſed conſiderably from its ſituation towards the left hypochondrium, and, beſides adhering firmly to the ſtomach, had its convex ſurface rendered almoſt concave, and was covered by a thick coat of coagulable lymph, diſcoloured by the bile, which had been ſo long in contact with it. The ſubſtance of the liver, however, appeared ſound.

There were numerous adheſions of the inteſtines to one another, and to the peritonæum, particularly on the left ſide, and, confined within them in ſeveral places, were ſmall quantities of a purulent fluid. Some pus was likewiſe found in the cavity of a portion of inteſtine on the right ſide: it adhered firmly to the peritonæum at the part where the trocar entered, and ſeemed to have been ſlightly wounded by it. In ſhort, the bilious fluid was contained in one large cavity, formed chiefly by the right hypochondrium, which had been greatly enlarged by the diaphragm's yielding to the preſ-

ſure of the fluid upwards, having aſcended as high as between the ſecond and third ribs, ſo as to diminiſh the cavity of the cheſt in a ſurpriſing manner. The peritonæum, ſurrounding the fluid, was every where covered with coagulable lymph, bearing ſome reſemblance to a diſtinct and regular cyſt.

The liver being next ſeparated from the diaphragm and ſurrounding adheſions, was taken out with a view to examine the under ſurface, and to inquire particularly into the ſtate of the gall bladder and biliary ducts; but all theſe were involved in ſuch a ſtate of confuſion, in conſequence of the adheſions to the ſtomach and neighbouring parts, that nothing ſatisfactory with regard to the exact place at which the injury had been received could be aſcertained.

*Remarks.*

The foregoing caſe and diſſection appear to me highly worthy of being recorded; for as a great number of anomalous caſes occur in the practice of phyſic, and as they frequently throw light on each other, the faithful narration of any one of them may be conſidered an uſeful addition to the ſtock already obtained: and although though the knowledge of the preſent caſe ſhould not enable us to perform a cure in any ſimilar inſtance, yet, if it ſhould lead to a more accurate prognoſis, or if it ſhould tend to illuſtrate or confirm any points in phyſiology or pathology, which were before doubtful, it may ſtill be rendered ſubſervient to a valuable purpoſe.

The diſcharge of a bilious fluid by the operation of the paracenteſis is not a new obſervation, ſince, in caſes of dropſy, complicated with jaundice, ſomething ſimilar has been noticed; but the proportion of bile at ſuch times is commonly ſmall, the ſerum only being coloured with it; and we are not at a loſs how to account for ſuch an effuſion. I have not been able to learn, however, that any caſe of the ſame nature as the preſent has been noticed by medical writers. It was rendered particularly intereſting by the length of time which the patient ſurvived the accident, as it generally happens, when external injuries affecting the viſcera prove fatal, that death is ſoon induced in conſequence of profuſe hæmorrhage from the rupture of the ſpleen, liver, or other viſcera, or of the ſpeedy termination of acute inflammation in gangrene.

The diffection is not fo complete as could be wifhed, fo that it muft remain a doubt whether the bile was effufed into the cavity of the abdomen from a rupture of the gall bladder, or of the biliary ducts. If we fuppofe from the former, we can fee no reafon why fome bile fhould not have paffed into the duodenum through the hepatic duct and ductus communis; and in that cafe the fæces fhould have affumed more of the natural appearance; and, on the other hand, fhould we refer the injury to the ducts themfelves, we are equally at a lofs to underftand why the vena portarum and hepatic artery, which are alfo inclofed in Gliffon's capfule, fhould have efcaped entire. But whatever may be faid in favour of either of thefe fuppofitions, no perfon, I imagine, will hefitate to admit that the bile, when fecreted by the liver, was effufed through one or other of the channels which have been mentioned into the cavity of the abdomen. This was conjectured immediately after the firft tapping, and was indeed the moft obvious, if not the only, explanation which could be offered of fo uncommon an appearance.

There was every reafon, before he was tapped, to imagine that the diftention of the abdomen domen depended on a watery accumulation, as the ſymptoms of inflammation had been well marked, and as this condition had been frequently known to lead to very obſtinate dropſies, particularly of the cheſt; the increaſed action of the veſſels, in the firſt place, and the relaxation conſequent upon ſuch a ſtate of excitement in the ſecond, giving riſe to an effuſion conſiderably greater than that which takes place from the extremities of the exhalent arteries in health. That ſuch an increaſed exhalation was in part the cauſe of the enlargement cannot be doubted, when we conſider that ſixteen pints of pure bile could never have been furniſhed by the liver in ſo ſhort a ſpace of time.

With regard to the adheſions, it is probable that they were all formed after the period of the firſt operation, as the fluctuation was at that time evident in every part, and the fluid readily and completely evacuated by an opening on the left ſide in the uſual place. The explanation which I would ſuggeſt is, that a ſlight inflammation of the peritonæum was produced ſoon after the tapping, and that adheſions were formed for the purpoſe of putting a ſtop to it, a uſe which, in a variety of inſtances, they ſeem effectually effectually to perform. In ſuch patients as die conſequent upon the operation of the paracenteſis, which are more numerous, I believe, than is commonly ſuſpected, we can generally trace circumſtances unfavourable to the proceſs of adheſion; ſuch as a diſeaſed condition of the viſcera, or the utmoſt ſtate of debility from the frequent returns of the diſeaſe, by which the peritonæum itſelf is in ſome caſes much diſeaſed likewiſe, and unequal to the phænonena of healthy inflammation.

The favourable ſymptoms attending this patient the firſt and ſecond days after the firſt operation, led me to conclude, rather haſtily, that adheſions had taken place in ſuch a manner as to prevent a farther effuſion. I was not then aware that the conſtant diſcharge of bile from the ruptured orifice was ſufficient to render the adheſion of it impracticable.

The appearances on diſſection authoriſe us to conclude that the adheſions were formed at the part the fartheſt from the ſource of the effuſion, viz. on the left ſide and lower part of the abdomen, and hence the diſtinct cavity in which the bilious fluid was contained. It muſt be allowed at the ſame time, that the ſecond attempt to tap the patient gave riſe to freſh inflammation, flammation, and to a farther depoſit of coagulable lymph.

It is a remarkable circumſtance that the bile, when preſented to ſo large a ſurface, ſhould not have been more freely abſorbed, ſo as to impregnate the whole maſs of circulating fluids. If a farther confirmation of the obſervation, that inflamed ſurfaces are unfavourable to abſorption, be required, the preſent inſtance may be adduced as a ſtriking illuſtration of it.

The effect of the bliſter in diminiſhing or ſuſpending the inflammation deſerves to be noticed, and ſhould, I think, teach us never to neglect ſo important a remedy when we ſuſpect its preſence in the peritonæum or any of the abdominal viſcera.

There is one additional remark which the preſent caſe cannot fail to ſuggeſt. It would, in my opinion, juſtify ſurgeons in making an opening with the trocar, in doubtful caſes, wherever the fluctuation is moſt evident. According to this idea, the part to have tapped this boy with ſucceſs would have been midway between the navel and laſt rib on the right ſide, inſtead of midway between the former and ſpinous proceſs of the os ilium on the left.

I cannot

I cannot conclude these obſervations without expreſſing my beſt acknowledgements to Mr. Cooper, one of the ſurgeons of Guy's hoſpital, who was preſent at the diſſection, and has kindly contributed every thing in his power ſince that time, to render the caſe accurate and inſtructive.

*Bow Lane, Cheapſide,*

August 10, 1785.

